# Immobilization of NTPDase-1 from* Trypanosoma cruzi* and Development of an Online Label-Free Assay

**DOI:** 10.1155/2016/9846731

**Published:** 2016-12-14

**Authors:** Felipe Antunes Calil, Juliana Maria Lima, Arthur Henrique Cavalcante de Oliveira, Christiane Mariotini-Moura, Juliana Lopes Rangel Fietto, Carmen Lucia Cardoso

**Affiliations:** ^1^Departamento de Química, Grupo de Cromatografia de Bioafinidade e Produtos Naturais, Faculdade de Filosofia Ciências e Letras de Ribeirão Preto, Universidade de São Paulo, 14040-901 Ribeirão Preto, SP, Brazil; ^2^Departamento de Bioquímica e Biologia Molecular, Universidade Federal de Viçosa, 36570-000 Viçosa, MG, Brazil; ^3^Instituto Nacional de Biotecnologia Estrutural e Química Medicinal em Doenças Infecciosas (INBEQMeDI), São Carlos, SP, Brazil

## Abstract

The use of IMERs (Immobilized Enzyme Reactors) as a stationary phase coupled to high performance chromatographic systems is an interesting approach in the screening of new ligands. In addition, IMERs offer many advantages over techniques that employ enzymes in solution. The enzyme nucleoside triphosphate diphosphohydrolase (NTPDase-1) from* Trypanosoma cruzi *acts as a pathogen infection facilitator, so it is a good target in the search for inhibitors. In this paper, immobilization of NTPDase-1 afforded ICERs (Immobilized Capillary Enzyme Reactors). A liquid chromatography method was developed and validated to monitor the ICER activity. The conditions for the application of these bioreactors were investigated, and excellent results were obtained. The enzyme was successfully immobilized, as attested by the catalytic activity detected in the* Tc*NTPDase-1-ICER chromatographic system. Kinetic studies on the substrate ATP gave *K*_*M*_ of 0.317 ± 0.044 mmol·L^−1^, which still presented high affinity compared to in solution. Besides that, the ICER was stable for 32 days, enough time to investigate samples of possible inhibitors, including especially the compound Suramin, that inhibited 51% the enzyme activity at 100 *µ*mol·L^−1^, which is in accordance with the data for the enzyme in solution.

## 1. Introduction

Neglected tropical diseases (NTDs) comprise a group of diseases caused by infectious and parasitic agents. NTDs such as Chagas disease, Sleeping Sickness, Leishmaniasis, and Schistosomiasis, among others, predominantly affect the poorest populations, threatening the lives of over one billion people worldwide, including half billion children [1]. Chagas disease, also known as American trypanosomiasis, is a potentially life-threatening illness caused by the protozoan parasite* Trypanosoma cruzi*. This disease occurs mainly in Latin America, where its transmission to humans happens via the feces of triatomine bugs, known as “kissing bugs” or some other names depending on the geographical area. Other modes of transmission include blood transfusion, congenital transmission, and organ transplants. According to the World Health Organization (WHO), Chagas disease affects between six and eight million people; about 12,000 deaths per year are a consequence of this disease. Roughly 65 million people are at risk of infection, and 200,000 new cases emerge each year.

Since the first clinical description of Chagas disease in 1909, several treatments have been tested; however, no safe and effective drug has been discovered. The currently available drugs have low efficacy in the chronic stage of the disease and cause severe side effects [2].

Nucleoside triphosphate diphosphohydrolases or apyrases from the CD39/GDa1 family (NTPDase, EC 3.6.1.5) belong to the group of hydrolases. These enzymes occur in several organisms, including* Toxoplasma gondii* [3],* T. cruzi* [4],* T. evansi* [5],* Trichomonas vaginalis* [6], and* Leishmania infantum* [7]. NTPDase from parasites can hydrolyze extracellular nucleoside tri- and diphosphate, thereby affecting the host's immune response [8–10]. So far, only one NTPDase has been described for* T. cruzi*, NTPDase-1, which is related to the virulence factor of this species. Inhibition of NTPDase-1 by partial enzymatic inhibitors or anti-NTPDase-1 antibodies lowers the level of* T. cruzi *adhesion, decreases host cell infection, and attenuates virulence in experimental infection [4, 11, 12]. A particular feature of these enzymes is the presence of five apyrase conserved regions (ACR), which consist of short sections of amino-containing residues that are essential for the enzyme to function [9]. This feature has made NTPDase-1 a target for the development of an alternative online assay in an attempt to discover new selective ligands.

ATP conversion to AMP or ADP aids kinetic studies and investigations into the effect of small molecules on the enzymatic activity of* Tc*NTPDase-1. This reaction could become an important tool to screen ligands during drug discovery.

Several assays have been proposed to monitor the ATP-hydrolyzing activity of ATP-dependent enzymes, including fluorimetric and calorimetric detection of ADP formation, ATP consumption [13], or inorganic phosphate (Pi) release [14] by coupled enzymes, and off-line liquid chromatography (LC) analyses [15, 16]. The colorimetric assay based on the malachite green reagent (MG) [17] allows for quantification of Pi released by the enzyme and is the most often employed method. Nevertheless, this assay is an indirect process that has some associated drawbacks: some target compounds and MG have similar wavelengths absorption; Pi contamination in the buffer can lead to erroneous results; and the use of high substrate concentrations is unfeasible.

Bioaffinity chromatography is one of the most versatile and promising alternatives to conventional assays. Interactions taking place in a bioaffinity column resemble the interactions that occur in many biological systems. The high specificity of these interactions provides high selectivity [18]. Immobilization of enzymes onto chromatographic supports has several advantages over the use of free enzymes in solution. For example, techniques based on immobilized enzymes (i) require small sample volumes and reduced sample handling, thus preventing contamination; (ii) provide the enzyme with increased lifetime and stability due to larger temperature control; (iii) allow for the use of a wider range of organic solvents and pH without significant loss of catalytic activity; (iv) enable easy separation of the enzyme and reaction products [19, 20] and (v) make enzyme reuse feasible.

Many enzymes from parasites underlying NTDs have been validated as targets in inhibition studies, which have pointed to the potential pharmacological application of the enzymes. For instance, the gGAPDH enzymes from* T. cruzi *[21] and humans [22] have been immobilized onto a fused silica capillary and used as a tool to screen inhibitors; the human enzyme was studied to evaluate specificity. Purine nucleoside phosphorylase (PNP) from* Schistosoma mansoni* (schistosomiasis) [23] and humans [24] have been immobilized which could be used as an alternative to the usual coupled assay for the selective screening of ligands [25]. Other authors [26] have immobilized the parasite itself; for example, the parasites* Trypanosoma brucei *(sleeping sickness) and* Leishmania major* (leishmaniasis) were immobilized onto a thermoreversible gel, and they could be used to evaluate inhibition of target enzymes.

This work reports on a new method to assess the activity and inhibition of NTPDase-1 from* T. cruzi*. The method consisted of a label-free assay that used an immobilized capillary enzyme reactor based on* Tc*NTPDase-1 (*Tc*NTPDase-1-ICER) coupled to a high performance liquid chromatography system, which allowed for enzymatic studies including kinetic analyses and the identification of ligands. To our knowledge, this is the first time that* Tc*NTPDase-1 has been covalently immobilized onto a capillary tube and that ATPase activity has been successfully monitored by an online method employing* Tc*NTPDase-1-ICER.

## 2. Material and Methods

### 2.1. Reagents and Chemicals

The enzyme* Tc*NTPDase-1 (EC 3.6.1.5, from* T. cruzi*) was expressed and purified as reported elsewhere [11] with the modifications already described [12]. Its substrates adenosine 5′-triphosphate di(tris) salt hydrate (ATP), adenosine 5′-diphosphate sodium salt (ADP), adenosine 5′-monophosphate disodium salt (AMP), guanosine 5′ triphosphate tris salt (GTP), and guanosine 5′-diphosphate tris salt from* Saccharomyces cerevisiae *(GDP) were purchased from Sigma-Aldrich (St. Louis, MO, USA). Buffer components and all the chemicals used during the expression and purification, immobilization, and chromatographic procedures were acquired from Sigma, Merck (Darmstadt, Germany), and Acros (Geel, Belgium). The water used in all the preparations was obtained from a MILLI-Q® system (Millipore®, São Paulo, Brazil). The fused silica capillary (0.375 mm × 0.1 mm I.D) was acquired from Polymicro Technologies (Phoenix, AZ, USA). All the buffer solutions were filtered through 0.45 *μ*m membrane filters of nylon Millipore and degassed before use in the HPLC experiments.

Samples of purified enzyme solution were lyophilized. Briefly, the samples were pipetted into a glass vial and frozen with liquid nitrogen. Then, they were placed into an FTS LyoStar freeze-drier (Stone Ridge, NY). The temperature was initially set to −1°C, and the sample was held at this temperature for about 1 h before it was cooled to −35°C. Drying was conducted under a chamber pressure of 70 mTorr for 4 h. The lyophilized enzyme was stored in a freezer at −20°C. Before use, the enzyme was weighed, quantified, and resuspended in an immobilization buffer consisting of 50 mmol L^−1^ HEPES pH 8.0 and 0.3 mol·L^−1^.

### 2.2. Apparatus

The enzyme was immobilized by using an 11 Plus advanced single syringe pump with dual RS-232 from Harvard Apparatus (Holliston, USA).* Tc*NTPDase-1-ICER was placed in a Shimadzu HPLC system (Shimadzu, Kyoto, Japan) consisting of two LC 20AD pumps. One of the pumps contained an FCV-20ALvalve for low-pressure gradient, a UV-Vis detector (SPD-M20AV), autosampler equipment (SIL-20A), and a six-port and three-way switching Valco samplevalve (Supelco, St. Louis, MO, USA). Data were acquired on a Shimadzu CBM-20A system interfaced with a computer; the Shimadzu-LC Solutions (LC Solution 2.1) software (Shimadzu, Kyoto, Japan) was used. A microplate reader system (Elisa readers) Versa Max-Molecular Device (Silicon Valley, CA, USA) was used.

### 2.3. Buffers

*Buffer A*: Tris-HEPES (2-Amino-2-(hydroxymethyl)propane-1,3-diol-4-(2-hydroxyethyl)-1-piperazineethanesulfonic acid) 50 mmol·L^−1^, pH 8.0 containing 3 mmol·L^−1^ MgCl_2_ (magnesium chloride), 116 mmol·L^−1^ NaCl, (sodium chloride) and 5.4 mmol·L^−1^ KCl (potassium chloride).* Buffer B*: Tris-HEPES (250 mmol·L^−1^, pH 8.0) containing 15 mmol·L^−1^ MgCl_2_ 580 mmol·L^−1^ NaCl, and 27 mmol·L^−1^ KCl.* Buffer C*: 50 mmol·L^−1^ KH_2_PO_4_ (potassium phosphate monobasic) containing 80 mmol·L^−1^ NH_4_Cl (ammonium chloride) and 3 mmol·L^−1^ TBHS (tetrabutylammonium hydrogen sulfate), pH 4.7.* Buffer D*: 50 mmol·L^−1^ KH_2_PO_4_, pH 7.4.* Buffer E*: HEPES 50 mmol·L^−1^, pH 8.0 containing 300 mmol·L^−1^ NaCl.

### 2.4. Activity of Free* Tc*NTPDase-1

Assays were performed in 96-well microplates. A microplate reader system (Elisa readers) was used to measure the absorbance. To evaluate the protein phosphatase activity, the Malachite Green Colorimetric Assay was employed [27].

The Pi (free phosphate) calibration curve was constructed by adding 150 *μ*L of Buffer A and 10 *μ*L of standard KH_2_PO_4_ 1.5 mg·mL^−1^ in the first well and 80 *μ*L of Buffer A in the other wells, followed by serial dilution.

Enzymatic activity was evaluated by adding 16 *μ*L of Buffer B, 3 *μ*L of nucleotide 25 mmol·L^−1^ (ATP, ADP, GDP or GTP), 0.5 *μ*g of enzyme, and enough water to complete 80 *μ*L, which was followed by incubation at 37°C for 10 min.

For both the calibration curve and the enzymatic reaction, 80 *μ*L of HCl 0.2 mol·L^−1^ was added (in the second case, to stop the reaction). Next, 40 *μ*L of the colorimetric reagent (ammonium molybdate 86 mmol·L^−1^/malachite green 0.2% 1 : 3) was added. The mixture was incubated at room temperature for 10 min and read at 650 nm. Analyses were performed in triplicate.

### 2.5. Immobilization of* Tc*NTPDase-1

The enzyme solution was prepared by resuspending the purified and/or the lyophilized enzyme in the immobilization buffer to a final concentration of 0.03 mg·mL^−1^ *Tc*NTPDase-1-ICER was prepared by using the protocol that had been described for immobilization of the enzymes glyceraldehyde-3-phosphate dehydrogenase (GAPDH) [21, 22] and acetylcholinesterase (AChE) [28]. In summary, using a syringe pump at flow rate 130 *μ*L·min^−1^, the capillary (100 *μ*m I.D × 0.375 mm × 30 cm) was cleaned with 2.0 mL of a 2 mol·L^−1^ HCl solution, followed by 1.0 mL distilled water. This procedure removes impurities on the inner surface of capillary and it allows access to silanol groups [29]. After that, the capillary was dried at 95°C for 1 hour. Then 1.0 mL 3-aminopropyltrietoxysilane (10% v/v) solution in water was passed through the capillary, which was subsequently dried in an oven at 95°C. The capillary was stored overnight at room temperature.

A glutaraldehyde solution 1% (v/v) in* Buffer D* (2.0 mL) was passed through the aminopropylsilica (APS) capillary, in order to remove glutaraldehyde excess and thus avoid polymerization; the capillary tubing was rinsed with* Buffer D* (0.5 mL). The enzyme immobilization was performed by passing 0.5 mL of* Tc*NTPDase-1 (0.03 mg·mL^−1^) in* Buffer E* through the capillary, three times. When not in use, the* Tc*NTPDase-1-ICER was kept at 4°C with the two ends of the capillary tubing immersed in Buffer A. Immobilization yield was evaluated using a method previously described by Bradford [30]; for this the enzymatic solution was measured before and after immobilization process.

### 2.6. Chromatographic Conditions

*Tc*NTPDase-1-ICER was conditioned with Buffer A at a flow rate of 0.03 mL·min^−1^ and 25°C; the first dimension of the two-dimensional method was employed. Then,* Tc*NTPDase-1-ICER was coupled to the Phenomenex Luna C18 (100 Å 250 × 4.6 mm, 5 *μ*m) analytical column at a flow rate of 1.0 mL·min^−1^ and conditioned with Buffer C : MeOH (84 : 16 v/v) [15, 31]. UV detection was conducted at *λ* = 254 nm through a three-way switching sample valve, to form the two-dimensional system. The enzymatic reaction occurred in the first dimension (*Tc*NTPDase-1-ICER), and the analytical separation of the substrate and the enzymatic products (AMP, ADP and ATP, resp.) occurred in the second dimension (C18 analytical column).

### 2.7. Validation of the Method

With the use of appropriate solutions, the ADP and AMP calibration curves were constructed by plotting the peak area against the analyte concentration. Both samples were prepared in triplicate at the following concentrations: 700, 500, 400, 300, 200, 100, 50, and 25 *μ*mol·L^−1^. Aliquots (10 *μ*L) were injected into the empty capillary (30.00 cm) coupled to the two-dimensional system. The intra- and interday precision and accuracy of the method were obtained by analyzing quality control samples at three different concentrations: 12.5, 6.25, and 3.125 *μ*mol·L^−1^. Five samples of each concentration were prepared and analyzed on three nonconsecutive days. The acceptance criterion for the limit of quantification was that the precision of three samples should be under 20% variability. The limit of detection was calculated by taking a signal-to-noise ratio of 3. The parameters established by ANVISA (Brazilian National Agency of Sanitary Regulation) for bioanalytical methods [32, 33], selectivity, linearity, repeatability, precision, accuracy, limit of quantification (LOQ), and limit of detection (LOD) were evaluated

### 2.8. Kinetic Studies

#### 2.8.1. Assay on* Tc*NTPDase-1-ICER

The online activity of* Tc*NTPDase-1 was assayed by using the validated* Tc*NTPDase-1-ICER two-dimensional method. ATP stock solutions were prepared at concentrations of 0.5, 1, and 10 mmol·L^−1^. To perform the kinetic studies, 10 *μ*L of different concentrations of the substrate ATP (from 0.025 to 7 mmol·L^−1^) was prepared from a stock solution and injected in triplicate. Nonlinear regression analysis with the Sigma Plot software version 11.0 was used to determine *K*_*M*_ values.

### 2.9. Inhibition Studies

#### 2.9.1. Assay on Free* Tc*NTPDase-1

The inhibitory activity of two compounds, gadolinium chloride (GdCl_3_) and Suramin sodium salt, toward free* Tc*NTPDase-1, was evaluated. The assays were performed in 96-well microplates by adding 16 *μ*L of Buffer B, 3 *μ*L of ATP 25 mmol·L^−1^, 0.5 *μ*g of enzyme, 1 *μ*L of inhibitor sample 100 *μ*mol·L^−1^, and enough water to complete 80 *μ*L in each well. The plates were then incubated at 37°C for 10 min. Next, 80 *μ*L of HCl 0.2 mol·L^−1^ (to stop the reaction) and 40 *μ*L of colorimetric reagent (ammonium molybdate 86 mmol·L^−1^ malachite green 0.2% 1 : 3) were added to the wells, and the mixture was incubated at room temperature for 10 min and read at 650 nm. Analyses were performed in triplicate. Inhibition was calculated based on the decrease in activity.

#### 2.9.2. Assay on* Tc*NTPDase-1-ICER

The inhibitory activity of the target compounds toward* Tc*NTPDase-1-ICER was investigated. To this end, 10 *μ*L of the sample containing 1 mmol·L^−1^ of ATP and 100 *μ*mol·L^−1^ of the assessed compound were injected into the developed* Tc*NTPDase-1-ICER system at room temperature. The percent of inhibition was calculated by comparing the enzymatic activity recorded in the absence and in the presence of the target compound. The formula %  *I* = 100 − (*C*_*i*_/*C*_0_ × 100), where *C*_*i*_ is the product concentration obtained in the presence of the analyzed compound and *C*_0_ is the product concentration calculated in the absence of the evaluated compound, was employed. Furthermore, the effect of the analyzed compound on nucleotides separation was evaluated (Control Compound); it ensures that enzymatic activity can be measured in the presence of these compounds.

## 3. Results and Discussion

### 3.1. Immobilization

Immobilization process occur between the groups on enzyme surface and reactive groups in support. In this study, we used covalent immobilization by Schiff's base formation using glutaraldehyde as spacers; it is a common technique that has been applied to different supports [34]. This technique is based on the successful immobilization of enzymes onto fused silica capillary reported by our research group [21, 28]. Fused silica capillary was the support selected for NTPDase-1 immobilization. Previously reported conditions were used to construct* Tc*NTPDase-1-ICER.

**Figure 1:**
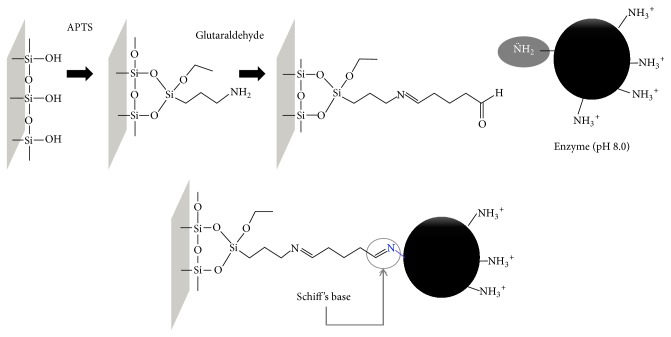
Representative scheme of enzyme immobilization via Schiff's base formation. Capillary support was activated with 3-aminopropyltrietoxysilane (APTS) and glutaraldehyde, subsequently N-terminal amino group of enzyme binding to aldehyde support.

Silica capillary tubing activated with aldehyde groups enables occurring immobilization with both N-terminal groups (pKa 7.5) and lysine residues (pKa 10.5). To ensure immobilization of* Tc*NTPDase-1* via* Schiff base formation between N-terminal amino group and aldehyde onto the support, we used pH 8.0 (*Buffer E*). In these conditions the amino groups of lysine residues are protonated and unavailable to bind to aldehyde (Figure 1). This procedure provided selective immobilization and minimized conformational changes in the structure of the enzyme [35]. Furthermore, the buffer used for immobilization (*Buffer E*) was selected based on the studies for free enzyme in solution; here we tried mimicking these conditions for conservation of catalytic properties of the target enzyme [12].

The immobilization yield was measured by Bradford method [30]; the enzymatic solution after immobilization process was evaluated and any significant amounts of residual enzyme were observed. These results allowed to suggest that immobilization yield was 100%. The enzyme was successfully immobilized on the capillary, and its enzymatic activity was retained.

### 3.2. *Tc*NTPDase-1-ICER Two-Dimensional Method

The bioreactor was not able to separate the substrate (ATP) from the products (ADP and AMP). Therefore, nucleotides were separated by ion-pair chromatographic using an isocratic elution in the second dimension. Ion pair chromatography (IPC) can be regarded as a modification of reverse phase chromatography (RPC). The only difference in conditions for IPC is the addition of an ion-pairing reagent (TBHS) to the mobile phase, which can then interact with ionized nucleotides in an equilibrium process [36]. The mobile phase composition and flow rate were optimized on the basis of literature chromatographic methods used to separate 5′-triphosphate and its reversed-phase by HPLC on porous stationary phases (C18) [15, 31]. This method should provide high selectivity and resolution values. Ion-pair chromatographic separations are sensitive to pH changes, and Buffer A was transferred from the first to the second dimension, so optimization of the coupled time was necessary. Because the mobile phase of the second dimension had no contact with* Tc*NTPDase-1-ICER, it was possible to use organic modifiers during isocratic ion-pair chromatographic separations safely.

**Figure 3:**
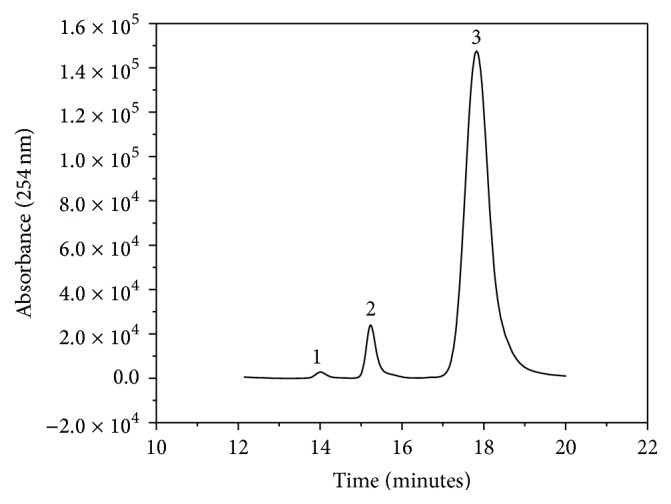
Representative chromatogram for the separation of ATP (3), ADP (2), and AMP (1) by a Phenomenex Luna C18 (100 Å 250 × 4.6 mm, 5 *μ*m) analytical column at a flow rate of 1.0 mL·min^−1^, eluent Buffer C : MeOH (84 : 16 v/v), and UV detection at *λ* = 254 nm. The chromatographic conditions of the two-dimensional method are described in Table 1.

Figure 3 evidences separation of the three nucleotides with good separation factors (*α*_1-2_ = 1.10 and *α*_2-3_ = 1.16) and retention times of 14 min, 15.5 min, and 18 min for AMP (1), ADP (2), and ATP (3), respectively. The bands were identified by injection of the standard solutions under the same chromatographic conditions and comparison of the retention times. This condition was used to validate the chromatographic method and to screen inhibitors.

### 3.3. Validation of the Chromatographic Method

To quantify the products, the area of the respective chromatographic band was employed, and the calibration curve was extrapolated. The method was validated, and calibration curves were constructed for ADP and AMP. The relationships between the injected concentrations and the peak areas were linear: *y* = 7748*x* − 9339, *r* ^2^ = 0.99921, for ADP and *y* = 7751*x* − 63084, *r* ^2^ = 0.99979, for AMP. Supporting Information (SI) Figures S1 and S2 (in Supplementary Material available online at http://dx.doi.org/10.1155/2016/9846731) contains more details about this issue. The intraday precision was expressed as the coefficient of variation (CV%); CV values less than or equal to 15% were accepted. Accuracy was determined as the ratio percentage between the average value provided by the validated method and the reference value for the added concentration. For accuracy, deviations less than or equal to 15% were accepted. The coefficient of variation (CV) afforded the intra- and the interday variability CV ranged from 0.22 to 12.1% with accuracy values ranging from 97.3 to 106.5% for ADP and from 0.21 to 3.02% with accuracy values ranging from 98.7 to 107.4% for AMP. The limit of quantification was 12.5 *μ*mol·L^−1^ (RSD = 1.99% with 83.5% accuracy) and 25 *μ*mol·L^−1^ (RSD = 2.02% with 106.8% accuracy) for ADP and AMP, respectively. The limit of detection of the method was calculated as 10 *μ*mol·L^−1^ and 15 *μ*mol·L^−1^ for ADP and AMP, respectively, for a signal/noise ratio equal to 3. Supporting Information (SI) Tables S1 and S2 contain additional details. The selectivity of the method was assured by analysis of blank samples before and after each sequence. The values of the evaluated parameters met the requirements of bioanalytical methods.

### 3.4. Determination of the Catalytic Activity and Stability of* Tc*NTPDase-1-ICER

**Figure 4:**
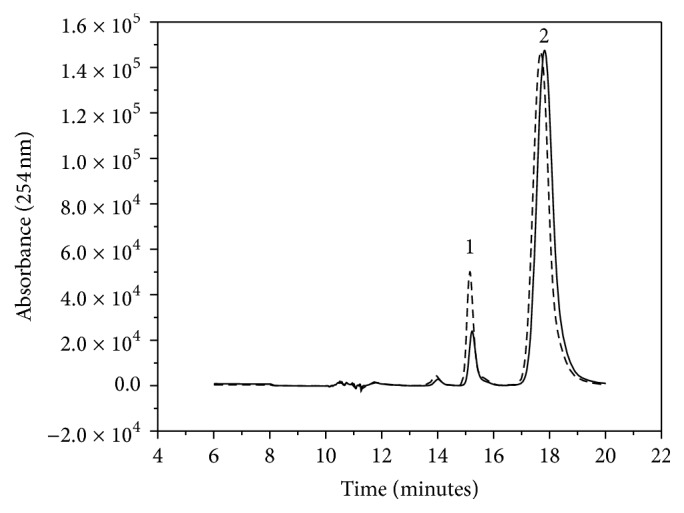
(—) Representative chromatogram of the separation of ATP (2) and ADP (1) by a Luna C18 analytical column. (- - -) Representative chromatogram of the activity of the immobilized enzyme* Tc*NTPDase-1-ICER. On-line hydrolysis of ATP (10 *μ*L of 1.0 mmol·L^−1^) to ADP. Separation obtained by a Luna C18 analytical column coupled to the* Tc*NTPDase-1-ICER, max = 254 nm. Experimental conditions as described in Table 1.

To monitor the enzymatic activity, the increase in the bands of the products ADP and AMP where substrate ATP was used (or GDP and GMP where GTP was used as substrate) was compared by means of the method validated for* Tc*NTPDase-1-ICER. The chromatographic bands were compared with the chromatographic bands obtained when an empty capillary was coupled to the chromatographic system and 10 *μ*L of ATP 1 mmol·L^−1^ was injected into it. As seen in Figure 4, the band with retention time of 15.5 min (corresponding to ADP) increased, which confirmed the activity of the immobilized enzyme.

A very interesting point to consider is the enzyme stability in* Tc*NTPDase-1-ICER. The enzyme retained 86% of its initial activity for up to 32 days, but it decreased thereafter. Although 32 days was not a long period, it was sufficient to perform various studies and to evaluate the kinetic mechanisms of inhibition. The great advantage of this system was the possibility to reuse the enzyme, which represented savings as compared with consumption of the fresh enzyme in solution. The immobilized enzyme also allowed for analytical reproducibility because it employed the same batch of enzyme repeated times. Storage of the enzyme in the lyophilized form was also assessed in solution and after immobilization. The enzymatic activity did not change. The activities depicted in Figure 3 were recorded for* Tc*NTPDase-1-ICER constructed from lyophilized* Tc*NTPDase-1 and for* Tc*NTPDase-1 resuspended in immobilization buffer. The maximum storage stability of the lyophilized enzyme was 180 days.

Although ATP and ADP have been described as substrate for NTPDases, these enzymes can hydrolyze different nucleotides as well as UTP or GTP [12]. The immobilization process can affect the catalytic site or it can cause conformational changes in the structure of the target enzyme. Here, the activity enzymatic for different nucleotides was evaluated: ATP, ADP, GTP, and GDP [12]. Results suggested that the specificity of enzyme remained unaltered after immobilization, being the same specificity as in solution: GDP > GTP > ADP > ATP. All assays were done by evaluating the enzymatic activity to each nucleotide individually monitored by the increase in the product chromatographic peak area as exemplified in Figure 3. ATP was the nucleotide selected for the other tests described below because it displays good activity and separation and it is far less expensive than the others.

### 3.5. *K*_*M*_ Determination for* Tc*NTPDase-1

The kinetic parameter *K*_*M*_ allows for determination of the binding affinity between the enzyme and the substrate. *K*_*M*_ corresponds to the concentration of substrate at which the reaction rate equals half the maximum catalysis velocity. The binding affinity between* Tc*NTPDase-1-ICER and the substrate ATP was determined and compared with literature results obtained for the enzyme in solution [12].

**Figure 5:**
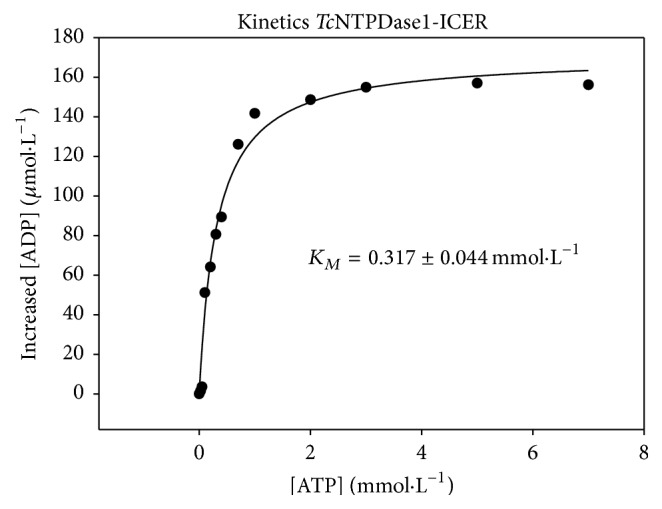
Michaelis-Menten hyperbole (*K*_*M*_) for* Tc*NTPDase-1-ICER.

In this experiment, increasing ATP concentrations (0.025 to 7 mmol·L^−1^) were used. The *K*_*M*_ value was 0.317 ± 0.044 mmol·L^−1^.  Figure 5 represents the Michaelis-Menten hyperbole.

At low concentrations of the substrate, the reaction rate was directly proportional to the concentration of the substrate. Progressive rise in the concentration of the substrate produced smaller increase in the reaction rate. Thus, the hyperbole profile agreed with the Michaelis-Menten equation. The *K*_*M*_ value found for the immobilized enzyme was 3.3 times higher than the *K*_*M*_ value reported for the enzyme in solution 0.317 and 0.096 *K*_*M*_, respectively [12]. This was expected because several factors can affect immobilized enzymes; for example, conformational change of the enzyme molecule due to changes in the tertiary structure of the active site; steric effects that can make the active site of the enzyme inaccessible; or diffusional mass transfer effects originating from resistant diffusion of the substrate and reaction product to and from the catalytic site of the enzyme, respectively [31]. It is worth noting that enzymes in solution are under static conditions, whereas in the proposed method they are under a flow system. Nevertheless, the enzymatic activity and selectivity were maintained in* Tc*NTPDase-1-ICER, which should enable the use of the proposed method to screen inhibitors.

### 3.6. Inhibition Studies

Inhibition studies were performed for* Tc*NTPDase-1-ICER to evaluate application of the immobilized enzyme in the screening of inhibitors. The results obtained for* Tc*NTPDase-1-ICER were compared with the results from the inhibition assay conducted for* Tc*NTPDase-1 in solution. Two known NTPDase compounds were already tested; Suramin and gadolinium chloride were tested. Suramin has previously been demonstrated to inhibit recombinant* Tc*NTPDase-1. Gadolinium can inhibit in vivo ectonucleotidase activity in* T. cruzi*, but it cannot inhibit the recombinant* Tc*NTPDase-1 [11].

**Figure 6:**
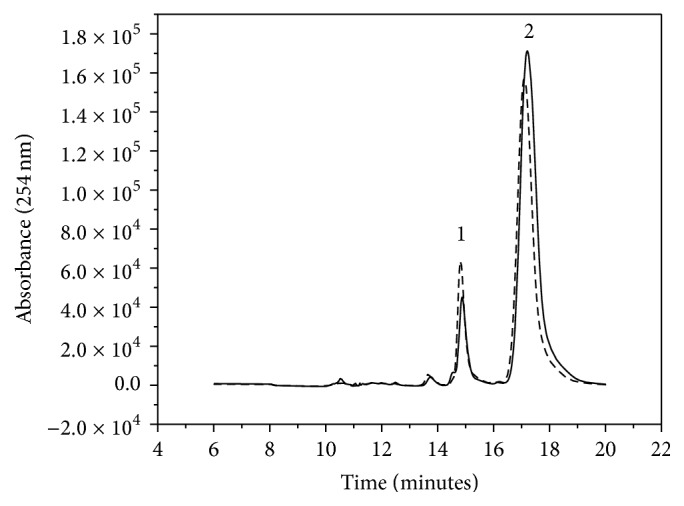
Representative chromatogram of the reduction in the activity of* Tc*NTPDase-1-ICER in the presence of 100 *μ*mol·L^−1^ Suramin (—). Representative chromatogram in the absence of Suramin (- - -). Chromatographic conditions as described in Table 1.

The ATP concentration of 1 mmol·L^−1^ (±3.15 times the *K*_*M*_ value) used in this work ensured saturation and conditions of initial velocity (*v*_0_) of the immobilized enzyme. The presence of a known concentration of Suramin reduced the band area of the hydrolysis substrate. More specifically, Sumarin showed 51% of inhibition. Our data agreed with the results from the inhibition tests conducted for the enzyme in solution - 69% in the presence of Suramin. Also, gadolinium chloride did not inhibit the enzyme, as expected.  Figure 6 illustrates the decrease in the absorption band of the product ADP (15.5 min) in the presence of 100 *μ*mol·L^−1^ Suramin.

## 4. Conclusions

Expression and purification methods were efficient and produced the purified enzyme with activity in an optimized refolding buffer; stability lasted up to 90 days in solution. In addition, aliquots of the purified enzyme previously submitted to lyophilisation retained the enzymatic activity after direct resuspension in the immobilization buffer as well as after immobilization onto fused silica capillary.* Tc*NTPDase-1 was successful, and the activity and stability of this enzyme were evaluated by the two-dimensional* Tc*NTPDase-1-ICER method. The ICER was stable for approximately 32 days, which was enough to investigate several samples of possible inhibitors. It was demonstrated to be an efficient tool for evaluating the enzyme activity and kinetic parameters, allowing future important studies involving ligand-protein interactions. The method was automated; the analyses required a small amount of samples. Additionally, the device was sensitive to the known* Tc*NTPDase-1 inhibitor Suramin. Based on the tests conducted with candidate inhibitors of the enzyme, this method is a promising tool for use in studies of selective inhibitors of* Tc*NTPDase-1 and represents an alternative strategy to perform kinetic studies for enzyme characterization.

## Supplementary Material

Fig. S1. ADP Calibration Curve. Fig. S2. AMP Calibration Curve. Table S1. Intra-day precision (n = 5) and method accuracy for ADP quantification. Table S2. Intra-day precision (n = 5) and method accuracy for AMP quantification.

## Figures and Tables

**Figure 2:**
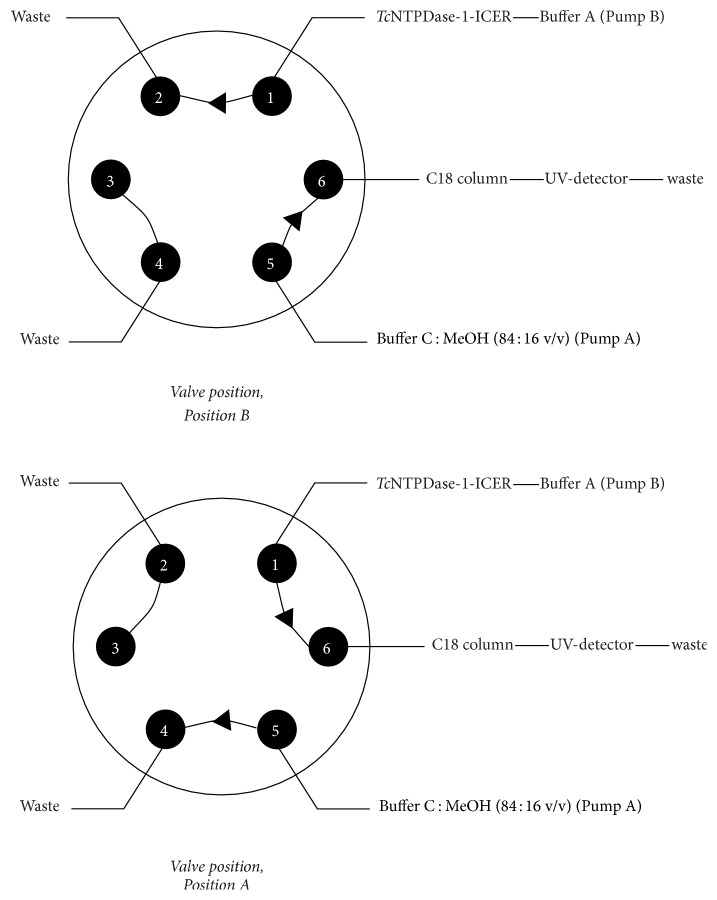
Schematic diagram of the two-dimensional system and the switch valve positions. Position B: column conditioning; Position A: coupled columns. In the chromatographic conditions optimized for the two-dimensional system (Figure 1), Phenomenex Luna C18 (100 Å 250 × 4.6 mm, 5 *μ*m) analytical column conditioned with Buffer C : MeOH (84 : 16 v/v) at a flow rate of 1.0 mL·min^−1^ and UV detection at *λ* = 254 nm led to the best analytical separation of ATP, ADP, and AMP (Figure 2).  Table 1 summarizes all the optimized conditions.

**Table 1:** Chromatographic conditions used in the *Tc*NTPDase-1-ICER two-dimensional method.

Pump	Time (min)	Valve position	Event
A	0.0–0.3	1	Enzymatic reaction in *Tc*NTPDase-1-ICER
B	0.0–0.5	1	Analytical column conditioning
A	0.31–8.0	2	Analyte transfer to the analytical column
A	8.01–20.0	1	*Tc*NTPDase-1-ICER conditioning
B	8.01–20.0	1	Analytical separation of the analytes in the second dimension

Pump A: flow rate: 0.03 mL·min^−1^, eluent: Buffer A. Pump B: flow rate: 1.0 mL·min^−1^, eluent: Buffer C : MeOH (84 : 16; v/v).
