# Supplementary material for: Immobilization of NTPDase-1 from Trypanosoma cruzi and Development of an Online Label-Free Assay
Source: J Anal Methods Chem. 2016 Dec 14;2016:9846731. doi: 10.1155/2016/9846731 (PMC5192316; doi:10.1155/2016/9846731)
Supplement: Supplementary file 1 — Fig. S1. ADP Calibration Curve. Fig. S2. AMP Calibration Curve. Table S1. Intra-day precision (n = 5) and method accuracy for ADP quantification. Table S2. Intra-day precision (n = 5) and method accuracy for AMP quantification. [file 9846731.f1.pdf]

Immobilization of NTPDase-1 from *Trypanosoma cruzi* and development of an online label free assay

Felipe Antunes Calil,[a] Juliana Maria Lima,[a] Arthur. H. C. Oliveira,[a] Christiane Mariotini-Moura,[bc] Juliana Lopes Rangel Fietto,[bc] Carmen. Lucia. Cardoso.[a*]

Supporting information (SI)

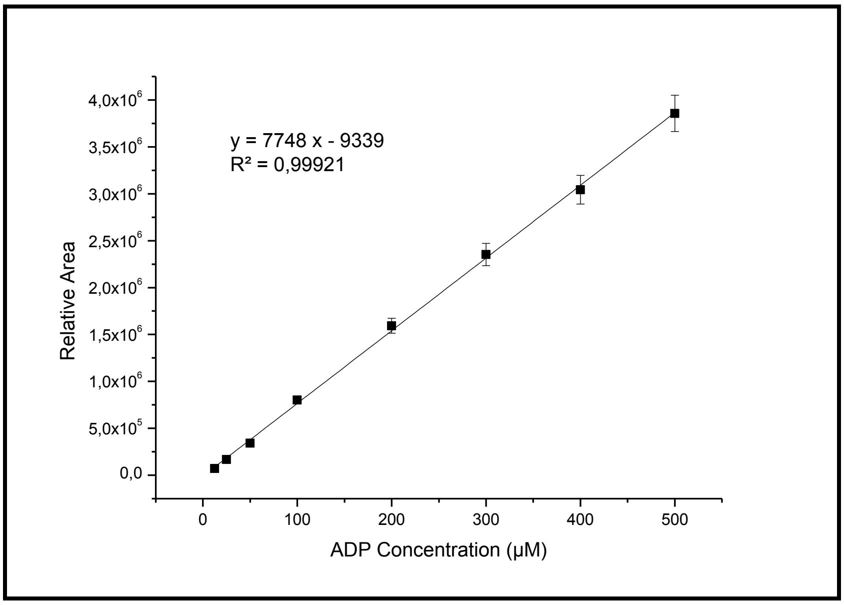

Fig. S1. ADP Calibration Curve.

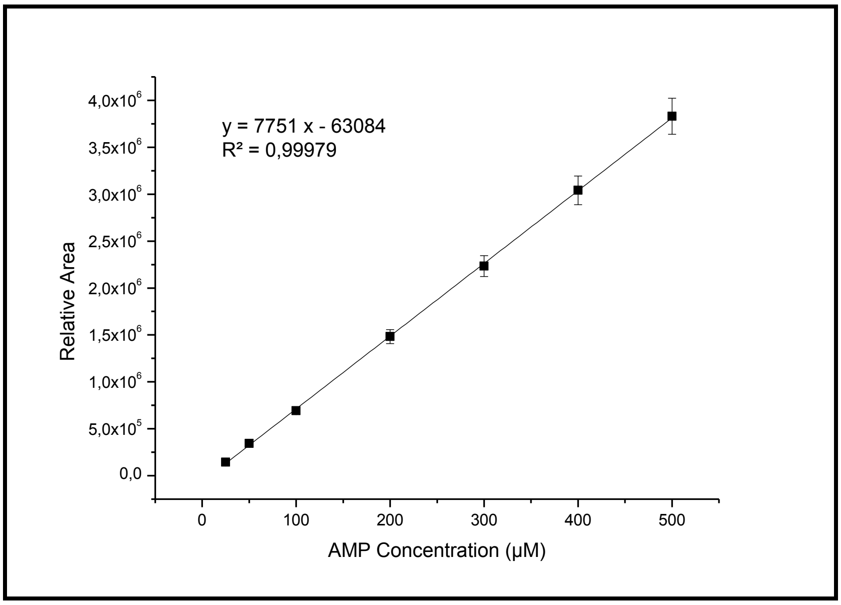

Fig. S2. AMP Calibration Curve.

Table S1. Intra-day precision (n = 5) and method accuracy for ADP quantification.

ADP Concentration (µmol/L)	*1st day*		*2nd day*		*3rd day*		*3-day mean*	
	CV (%)	Accuracy (%)	CV (%)	Accuracy (%)	CV (%)	Accuracy (%)	CV (%)	Accuracy (%)
40	6.83	109.0	4.11	104.0	4.30	106.5	5.08	106.5
250	0.63	96.7	0.41	98.4	0.75	96.9	0.60	97.3
450	0.22	97.3	0,51	98.9	5.01	96.6	1.91	97.6

Table S2. Intra-day precision (n = 5) and method accuracy for AMP quantification.

AMP Concentration (µmol/L)	*1st day*		*2nd day*		*3rd day*		*3-day mean*	
	CV (%)	Accuracy (%)	CV (%)	Accuracy (%)	CV (%)	Accuracy (%)	CV (%)	Accuracy (%)
40	3.02	111.7	1.07	110.7	1.65	99.7	1.91	107.4
250	0.07	101.9	0.19	98.2	0.31	95.5	0.19	98.5
450	0.21	101.1	0.46	99.5	0.13	95.4	0.27	98.7
